# Protein C activity may reflect endogenous anticoagulant balance and is associated with stroke severity and functional outcomes in acute ischemic stroke

**DOI:** 10.3389/fnagi.2026.1872982

**Published:** 2026-08-07

**Authors:** Soo-Hyun Park, Minwoo Lee, Yeo Jin Kim, Dongwon Lee, Jong Seok Bae, Ju-Hun Lee, Sang-Hwa Lee, Chulho Kim, Yerim Kim

**Affiliations:** 1 Department of Neurology, SoonChunHyang University, Seoul, Republic of Korea; 2 Department of Neurology, Hallym University Sacred Heart Hospital, Anyang-si, Gyeonggi-do, Republic of Korea; 3 Department of Neurology, Kangdong Sacred Heart Hospital, Hallym University College of Medicine, Seoul, Republic of Korea; 4 Department of Neurology, Chuncheon Sacred Heart Hospital, Hallym University College of Medicine, Chuncheon-si, Gangwon-do, Republic of Korea

**Keywords:** acute ischemic stroke, anticoagulant pathway, atrial fibrillation, cardioembolism, functional outcome, protein C, stroke severity, thrombosis

## Abstract

**Background:**

Aging is associated with a shift toward a prothrombotic state, partly driven by alterations in endogenous anticoagulant pathways. Protein C is a key component of this pathway, but the clinical relevance of variation in protein C activity for ischemic stroke has not been fully established. We investigated whether variation in protein C activity is associated with stroke phenotypes, stroke severity, and functional outcomes in acute ischemic stroke (AIS).

**Methods:**

We analyzed a prospective registry of patients with AIS admitted between 2016 and 2021. Protein C activity measured at admission was categorized into quartiles. Associations with stroke phenotypes, moderate-to-severe stroke (NIHSS ≥5), and poor 3-month functional outcomes (mRS ≥ 3) were evaluated using multivariable logistic regression. Additional analyses assessed the role of atrial fibrillation (AF) and potential effect modification by age.

**Results:**

Lower protein C activity was associated with a higher prevalence of AF and cardioembolic stroke. The proportion of moderate-to-severe stroke decreased across increasing quartiles of protein C activity (p for trend <0.001). Poor functional outcome also differed across protein C activity quartiles (*p* < 0.001). In multivariable analyses, higher protein C activity was independently associated with lower odds of moderate-to-severe stroke (OR 0.63 per 1-SD increase) and poor functional outcome (OR 0.71). The association between protein C activity and cardioembolic stroke was markedly attenuated and no longer statistically significant after adjustment for AF. No significant interaction between age and protein C activity was observed for moderate-to-severe stroke.

**Conclusion:**

Protein C activity was consistently associated with stroke severity and may reflect endogenous anticoagulant balance. An association with functional outcome was also observed, although this finding was less consistent across sensitivity analyses. These findings suggest that variation in protein C activity across its observed distribution may provide information that is not fully reflected by conventional laboratory reference categories.

## 1 Introduction

The endogenous anticoagulant system plays a critical role in regulating thrombin generation and maintaining hemostatic balance. Among these pathways, protein C serves as a key physiological inhibitor of coagulation through the inactivation of factors Va and VIIIa after activation by the thrombin–thrombomodulin complex (Anzola et al., 1993; Esmon, 2003). Dysregulation of endogenous anticoagulant pathways can therefore contribute to prothrombotic states and thromboembolic disease.

Although disturbances in the protein C pathway are well established in venous thromboembolism, their relevance in arterial thrombosis, including ischemic stroke, remains less clearly defined. Early clinical observations suggested that abnormalities in natural anticoagulant pathways may contribute to ischemic stroke, particularly in younger individuals (Hart and Kanter, 1990; Camerlingo et al., 1991; Majid et al., 2020). However, subsequent population-based studies have produced inconsistent findings, and large cohort investigations have not consistently demonstrated a strong association between isolated protein C abnormalities and arterial stroke risk (Zakai et al., 2018; Lefebvre et al., 1998; Lipe and Ornstein, 2011). Consequently, routine testing for inherited thrombophilia, including protein C deficiency, is generally not recommended in most patients with ischemic stroke (Morris et al., 2010; Hankey et al., 2001; Bushnell and Goldstein, 2000).

Most prior studies have focused on categorical definitions of protein C deficiency, which may overlook clinically meaningful variation across the spectrum of protein C activity. Because the protein C pathway regulates thrombin generation within the endogenous anticoagulant system, variation in protein C activity may reflect broader differences in endogenous anticoagulant activity and thrombogenic balance even in the absence of overt deficiency (Zakai et al., 2018; Folsom et al., 2009).

Aging is associated with a shift toward a prothrombotic state, characterized by alterations in both coagulation and endogenous anticoagulant pathways (Franchini, 2006; Mari et al., 2008; North and Sinclair, 2012). However, whether variation in protein C activity reflects age-related thrombogenic changes or represents a more fundamental component of anticoagulant balance across different age groups remains unclear.

The clinical significance of variation in protein C activity in acute ischemic stroke remains incompletely understood. We therefore investigated its associations with stroke phenotypes, stroke severity, functional outcomes, and potential effect modification by age.

## 2 Materials and methods

### 2.1 Study population

We analyzed a prospective registry of consecutive patients with AIS who were admitted between June 2016 and May 2021. Although the institutional stroke registry prospectively enrolls patients with both AIS and transient ischemic attack (TIA), only patients with AIS were eligible for inclusion in the present study. Patients with TIA and other non-AIS diagnoses were excluded before cohort selection. Patients were enrolled in the institutional stroke registry within 7 days of symptom onset and were managed according to standard stroke care protocols.

Among the initial cohort of 1,128 patients with AIS, 45 patients without available protein C activity measurements were excluded, leaving a final analytic cohort of 1,083 patients.

### 2.2 Clinical variables and outcomes

Baseline demographic and clinical information was obtained from the stroke registry, including age, sex, and vascular risk factors such as hypertension, diabetes mellitus, dyslipidemia, smoking history, and atrial fibrillation. Stroke etiology was classified according to the Trial of ORG 10172 in Acute Stroke Treatment (TOAST) criteria (Adams et al., 1993).

Stroke severity at admission was assessed using the National Institutes of Health Stroke Scale (NIHSS), with moderate-to-severe stroke defined as NIHSS ≥5 (Brott et al., 1989).

Functional outcomes were assessed at 3-month after stroke onset using the modified Rankin Scale (mRS). Poor functional outcome was defined as mRS scores of 3–6 (Banks and Marotta, 2007). Among the study population, 865 patients had available 3-month mRS data for outcome analysis.

### 2.3 Measurement of protein C activity

Although patients were enrolled within 7 days after symptom onset, blood samples for protein C activity were obtained immediately upon hospital admission according to the institutional stroke protocol. Protein C activity was measured using a chromogenic assay on an automated coagulation analyzer according to the manufacturer’s instructions. The reference range for protein C activity in our laboratory was 65–135 IU/dL (Gupta et al., 2022; Martinez et al., 1993).

For the primary analysis, patients were categorized into quartiles according to protein C activity (Q1: 15–86 IU/dL, Q2: 87–103 IU/dL, Q3: 104–119 IU/dL, and Q4: 120–200 IU/dL).

### 2.4 Statistical analysis

Baseline characteristics were compared across quartiles of protein C activity. Continuous variables were expressed as mean ± standard deviation (SD) and compared using ANOVA or the Kruskal–Wallis test, and categorical variables were compared using the chi-square test.

Multivariable logistic regression models were used to evaluate the associations of protein C activity with moderate-to-severe stroke (baseline NIHSS ≥5) and poor functional outcome (3-month mRS ≥ 3). Models evaluating stroke severity were adjusted for age, sex, hypertension, diabetes mellitus, stroke subtype (TOAST classification), coronary heart disease history, dyslipidemia, smoking status, atrial fibrillation, hematocrit, blood urea nitrogen, and C-reactive protein (Franchini, 2006). For models evaluating poor functional outcome, baseline NIHSS was additionally included as an adjustment covariate. Ordinal logistic regression (proportional odds model) using the full seven-level modified Rankin Scale (0–6) at 3-month was also performed. The proportional-odds assumption was evaluated and was not violated. Tests for linear trend across quartiles were conducted by entering the quartile variable as an ordinal variable in regression models.

Protein C activity was also analyzed as a continuous variable. Restricted cubic spline models with four knots placed at the 5th, 35th, 65th, and 95th percentiles of protein C activity were fitted to evaluate potential nonlinear associations with clinical outcomes. The spline models were adjusted for the same covariates as the primary multivariable analyses, and the median protein C activity of the study population was used as the reference value (odds ratio = 1).

Several additional sensitivity analyses were performed to evaluate the robustness of the primary findings. These included: (1) additional adjustment for reperfusion therapy (intravenous thrombolysis and/or endovascular thrombectomy); (2) exclusion of patients receiving pre-admission anticoagulant therapy; (3) multivariable models without adjustment for TOAST stroke subtype; (4) additional adjustment using laboratory reference categories of protein C activity (<65, 65–135, and >135 IU/dL); (5) ordinal logistic regression (proportional odds model) using the full seven-level mRS (0–6) at 3-month; and (6) inverse probability weighting (IPW) analysis to assess the potential influence of incomplete follow-up.

For the IPW analysis, the probability of having available 3-month mRS data was estimated using a multivariable logistic regression model including age, protein C activity, baseline NIHSS score, hematocrit, blood urea nitrogen, C-reactive protein, sex, hypertension, diabetes mellitus, dyslipidemia, smoking history, atrial fibrillation, and previous anticoagulation use. Stabilized inverse probability weights were calculated from the estimated probabilities and applied to weighted multivariable logistic regression models using the same covariate adjustment as in the primary analyses. The distribution of the stabilized weights was examined to assess the presence of extreme weights.

Additional secondary analyses were performed to evaluate the association between protein C activity and cardioembolic stroke before and after adjustment for atrial fibrillation, as well as the association between protein C activity and stroke severity after inclusion of atrial fibrillation in the multivariable model.

Potential effect modification by age was evaluated using predefined thresholds of 65 and 75 years. Interaction terms between continuous protein C activity and age were tested in the multivariable models, followed by age-stratified analyses.

Baseline characteristics were additionally compared between patients with and without available 3-month mRS data to evaluate potential bias related to incomplete follow-up.

Adjusted odds ratios (ORs) with 95% confidence intervals (CIs) were reported. All statistical tests were two-sided, and *p* < 0.05 was considered statistically significant.

All statistical analyses were performed using SPSS version 19.0 (IBM Corp., Armonk, NY, USA), except for the restricted cubic spline analyses, which were performed using Python version 3.13.5 with the statsmodels (version 0.14.6) and patsy (version 1.0.2) packages.

## 3 Results

### 3.1 Baseline characteristics

Among 1,083 patients with AIS included in the analysis, baseline characteristics according to quartiles of protein C activity are presented in Table 1. Patients with lower protein C activity were older and had different distributions of stroke subtypes across quartiles.

**Table 1:** Baseline characteristics according to quartiles of protein C activity.

Characteristics	Protein C activity, quartile 1 (*n* = 276)	Protein C activity, quartile 2 (*n* = 259)	Protein C activity, quartile 3 (*n* = 275)	Protein C activity, quartile 4 (*n* = 273)	*p*-value
Age, mean ± SD	73.2 ± 13.6	70.5 ± 12.7	66.2 ± 13.3	64.8 ± 12.9	<0.001
Female, *n* (%)	118 (42.8)	117 (45.2)	89 (32.4)	138 (50.5)	<0.001
TOAST classification, *n* (%)					<0.001
LAA	76 (27.5)	96 (37.1)	102 (37.1)	106 (38.8)	
SVO	40 (14.5)	50 (19.3)	73 (26.5)	73 (26.7)	
CE	80 (29.0)	40 (15.4)	35 (12.7)	30 (11.0)	
OD	13 (4.7)	11 (4.2)	12 (4.4)	18 (6.6)	
UD	67 (24.3)	62 (23.9)	53 (19.3)	46 (16.8)	
Vascular risk factor, *n* (%)					
Hypertension	190 (68.8)	159 (61.4)	186 (67.6)	177 (64.8)	0.163
Diabetes	89 (32.2)	87 (33.6)	101 (36.7)	109 (39.9)	0.325
Dyslipidemia	51 (18.5)	38 (14.7)	57 (20.7)	71 (26.0)	0.012
Smoking	64 (23.2)	63 (24.3)	116 (42.2)	86 (31.5)	<0.001
Atrial fibrillation	100 (36.2)	54 (20.8)	38 (13.8)	20 (7.3)	<0.001
Previous medication, *n* (%)					
Antiplatelet use	65 (23.6)	59 (22.8)	57 (20.7)	54 (19.8)	0.576
Anticoagulation use	34 (12.3)	6 (2.3)	4 (1.5)	6 (2.2)	<0.001
Warfarin	18 (6.5)	2 (0.8)	0 (0.0)	0 (0.0)	<0.001
Direct oral anticoagulants	16 (5.8)	4 (1.5)	4 (1.5)	6 (2.2)	0.004
Apixaban	4 (1.4)	0 (0.0)	1 (0.4)	2 (0.7)	–
Dabigatran	6 (2.2)	3 (1.2)	2 (0.7)	2 (0.7)	–
Rivaroxaban	3 (1.1)	0 (0.0)	1 (0.4)	2 (0.7)	–
Edoxaban	3 (1.1)	1 (0.4)	0 (0.0)	0 (0.0)	–
Reperfusion therapy (IV thrombolysis and/or endovascular thrombectomy), *n* (%)	57 (20.7)	48 (18.5)	30 (10.9)	19 (7.0)	<0.001
Stroke severity and outcome, *n* (%)					
Initial NIHSS, 0–4	134 (48.6)	171 (66.0)	209 (76.0)	207 (75.8)	<0.001
≥5	142 (51.4)	88 (34.0)	66 (24.0)	66 (24.2)	
3-month mRS, poor (mRS 3–6)	104/199 (52.3)	61/194 (31.4)	58/240 (24.2)	71/231 (30.7)	<0.001

TOAST, Trial of ORG 10172 in Acute Stroke Treatment; LAA, large artery atherosclerosis; SVO, small vessel occlusion; CE, cardioembolism; OD, other determined; UD, undetermined.

Values are presented as mean ± SD or *n* (%). Percentages were calculated using available data for each variable; therefore, denominators may vary because of missing values. Poor functional outcome was defined as a 3-month modified Rankin Scale score of 3–6.

Direct oral anticoagulants included apixaban, dabigatran, rivaroxaban, and edoxaban. No patients received heparin or low-molecular-weight heparin (LMWH) before admission.

**Figure 1:**
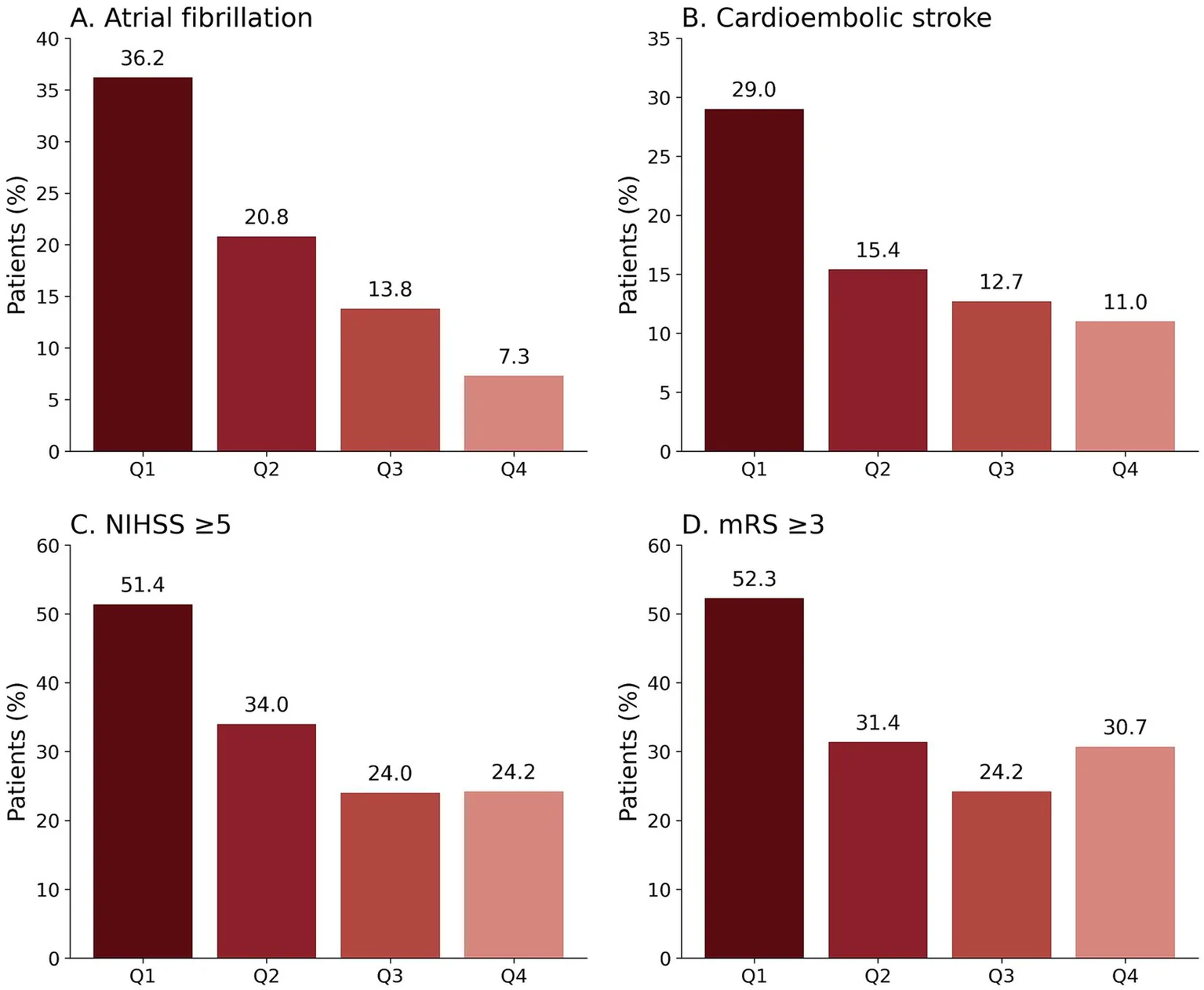
Association between protein C activity quartiles and stroke phenotypes in acute ischemic stroke. **(A)** Prevalence of atrial fibrillation. **(B)** Proportion of cardioembolic stroke. **(C)** Proportion of moderate-to-severe stroke (initial NIHSS ≥ 5). **(D)** Proportion of poor functional outcome at 3 months (mRS ≥ 3). Values represent percentages within each protein C activity quartile.

The prevalence of atrial fibrillation decreased progressively across increasing quartiles of protein C activity (36.2% in Q1, 20.8% in Q2, 13.8% in Q3, and 7.3% in Q4; *p* < 0.001) (Figure 1A). Similarly, the proportion of cardioembolic stroke decreased across quartiles (29.0, 15.4, 12.7, and 11.0%, respectively; p < 0.001) (Figure 1B).

### 3.2 Protein C activity and stroke severity

Stroke severity differed significantly across quartiles of protein C activity. The proportion of patients with moderate-to-severe stroke (NIHSS ≥5) was highest in the lowest quartile and decreased across higher quartiles (51.4, 34.0, 24.0, and 24.2%, respectively; p for trend <0.001) (Figure 1C).

**Figure 2:**
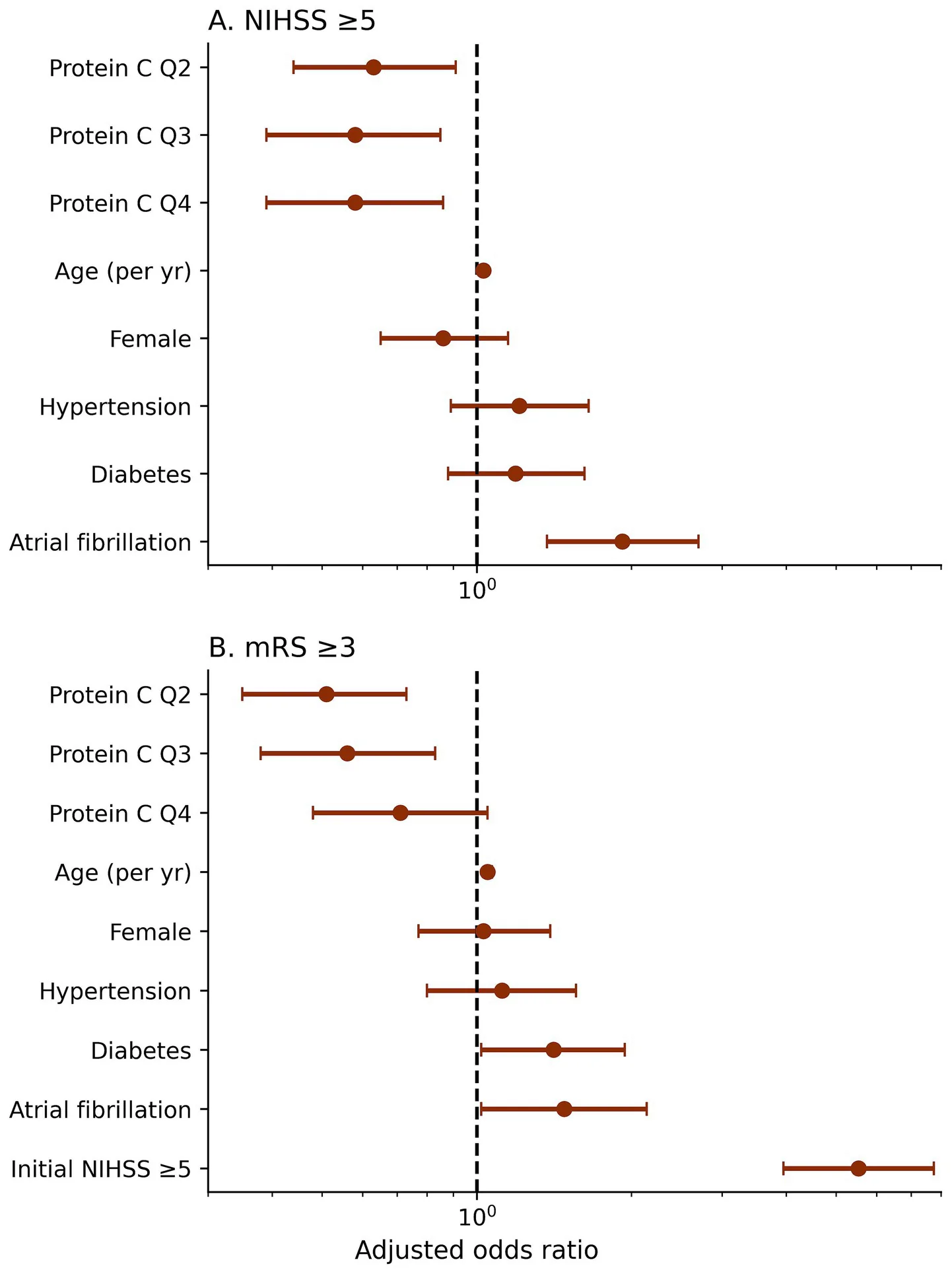
Multivariable associations of protein C activity quartiles with stroke severity and functional outcomes adjusted for clinically relevant covariates. Forest plots show adjusted odds ratios (*ORs*) and 95% confidence intervals (*CIs*) for **(A)** moderate-to-severe stroke (initial NIHSS ≥ 5) and **(B)** poor functional outcome at 3 months (mRS ≥ 3). Quartile 1 served as the reference category.

In multivariable logistic regression analysis, higher protein C activity remained independently associated with a lower risk of moderate-to-severe stroke (Figure 2). Compared with the lowest quartile, adjusted odds ratios were 0.63 (95% CI 0.44–0.91) for Q2, 0.58 (95% CI 0.39–0.85) for Q3, and 0.58 (95% CI 0.39–0.86) for Q4 (Table 2). Increasing age and atrial fibrillation were also independently associated with greater stroke severity.

**Table 2:** Association between protein C activity quartiles and stroke severity (NIHSS ≥5).

Variable	OR	95% CI	*p*-value
Protein C activity quartiles			
Q1	Reference		
Q2	0.63	0.44–0.91	0.014
Q3	0.58	0.39–0.85	0.005
Q4	0.58	0.39–0.86	0.007
Age (per year)	1.03	1.02–1.04	<0.001
Female (versus male)	0.86	0.65–1.15	0.310
Hypertension	1.21	0.89–1.65	0.220
Diabetes mellitus	1.19	0.88–1.62	0.260
Atrial fibrillation	1.92	1.37–2.70	<0.001

OR, Odds Ratio; CI, confidence interval; TOAST, Trial of ORG 10172 in Acute Stroke Treatment.

Adjusted for age, sex, hypertension, diabetes mellitus, stroke subtype (TOAST classification), coronary heart disease history, dyslipidemia, smoking status, atrial fibrillation, hematocrit, blood urea nitrogen, and C-reactive protein.

### 3.3 Protein C activity and functional outcomes

Functional outcomes at 3-month also differed across quartiles of protein C activity. The proportion of patients with poor functional outcome (mRS ≥ 3) was highest in the lowest quartile and lower in higher quartiles (52.3, 31.4, 24.2, and 30.7%, respectively; p for trend <0.001) (Figure 1D).

In multivariable analyses, higher protein C activity was associated with a lower risk of poor functional outcome (Figure 2). Compared with the lowest quartile, the adjusted odds ratios were 0.51 (95% CI 0.35–0.73) for Q2 and 0.56 (95% CI 0.38–0.83) for Q3, whereas the association for Q4 did not reach statistical significance (OR 0.71, 95% CI 0.48–1.05) (Table 3). Initial stroke severity was the strongest predictor of poor functional outcome (OR 5.53, 95% CI 3.95–7.75).

**Table 3:** Association between protein C activity quartiles and poor functional outcome (3-month mRS ≥ 3).

Variable	OR	95% CI	*p*-value
Protein C activity quartiles			
Q1	Reference		
Q2	0.51	0.35–0.73	<0.001
Q3	0.56	0.38–0.83	0.004
Q4	0.71	0.48–1.05	0.087
Age (per year)	1.05	1.04–1.07	<0.001
Female	1.03	0.77–1.39	0.840
Hypertension	1.12	0.80–1.56	0.510
Diabetes mellitus	1.41	1.02–1.94	0.037
Atrial fibrillation	1.48	1.02–2.14	0.038
Initial NIHSS ≥5	5.53	3.95–7.75	<0.001

OR, Odds Ratio; CI, confidence interval; NIHSS, National Institutes of Health Stroke Scale; TOAST, Trial of ORG 10172 in Acute Stroke Treatment.

Adjusted for age, sex, hypertension, diabetes mellitus, stroke subtype (TOAST classification), coronary heart disease history, dyslipidemia, smoking status, atrial fibrillation, hematocrit, blood urea nitrogen, C-reactive protein, and initial NIHSS.

### 3.4 Continuous and spline analyses

In continuous analyses, higher protein C activity was consistently associated with lower odds of adverse clinical outcomes. Each 1-SD increase in protein C activity was associated with a lower likelihood of moderate-to-severe stroke (OR 0.63, 95% CI 0.56–0.72; *p* < 0.001) and a reduced risk of poor functional outcome at 3-month (OR 0.71, 95% CI 0.61–0.82; *p* < 0.001) (Supplementary Table 1).

**Figure 3:**
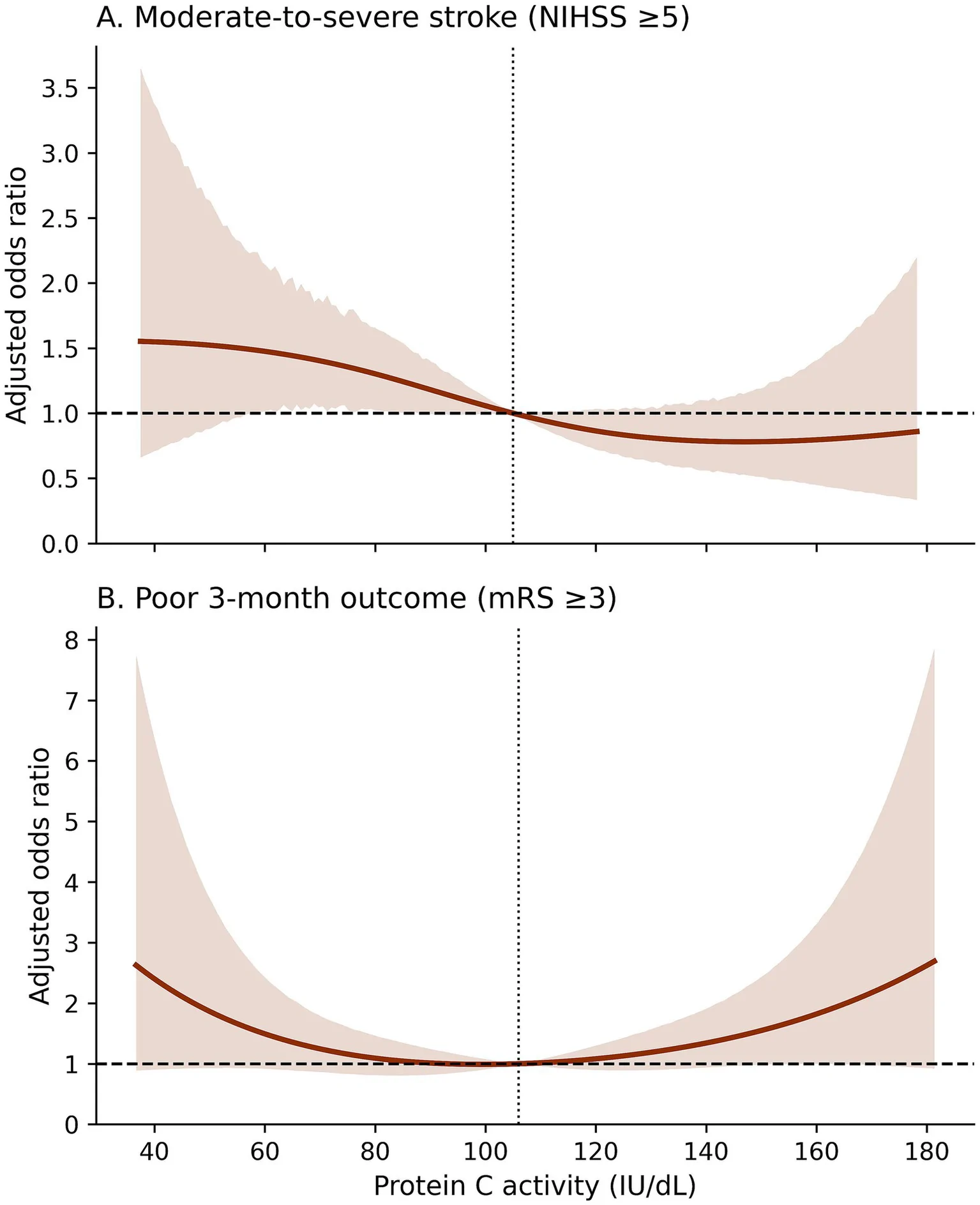
Restricted cubic spline analyses of the association between protein C activity and clinical outcomes. Restricted cubic spline models illustrating the adjusted association between protein C activity and **(A)** moderate-to-severe stroke (initial NIHSS ≥ 5) and **(B)** poor functional outcome at 3 months (mRS ≥ 3). Solid lines represent adjusted odds ratios, and shaded areas indicate 95% confidence intervals. The vertical dotted line indicates the median protein C activity (104.8 IU/dL), which was used as the reference value (*OR* = 1.0). A nonlinear association was observed for poor functional outcome but not for initial stroke severity.

Restricted cubic spline analyses demonstrated an inverse association between protein C activity and stroke severity, with progressively higher risk observed at lower protein C levels without evidence of a clear threshold effect. In contrast, a nonlinear association was observed between protein C activity and poor functional outcome (P for nonlinearity = 0.025), whereas the association with stroke severity remained approximately linear (P for nonlinearity = 0.97) (Figure 3).

### 3.5 Sensitivity analyses

Several sensitivity analyses were performed to evaluate the robustness of the primary findings. Sensitivity analyses generally supported the association between protein C activity and initial stroke severity, whereas the findings for functional outcome were less consistent across alternative model specifications (Supplementary Tables 2–13). Protein C activity showed consistent directional associations with stroke severity and functional outcomes (Supplementary Tables 2, 3).

Compared with patients with available 3-month mRS data, those without follow-up had lower protein C activity and higher proportions of female sex, hypertension, diabetes mellitus, and dyslipidemia, whereas age, baseline NIHSS, smoking history, atrial fibrillation, and previous anticoagulation use were similar between the groups (Supplementary Table 4).

In the IPW analysis, the second and third protein C activity quartiles remained significantly associated with lower odds of poor functional outcome, whereas the highest quartile was not significantly associated with poor functional outcome. Compared with the primary complete-case analysis, the continuous association between protein C activity and poor functional outcome was no longer statistically significant after IPW (OR, 0.99; 95% CI, 0.80–1.21; *p* = 0.898) (Supplementary Table 5).

To clarify the associations between protein C levels and clinical outcomes, we conducted sensitivity analyses accounting for reperfusion therapy, pre-stroke anticoagulant use, and TOAST classification.

Across the sensitivity analyses, higher protein C activity quartiles tended to be associated with lower initial stroke severity, although the specific quartiles reaching statistical significance varied across models (Supplementary Tables 6–8).

Among the 1,083 patients included in the primary analyses, 79 (7.3%) had protein C activity below the laboratory reference range (<65 IU/dL). In the sensitivity analysis using laboratory reference categories (<65, 65–135, and >135 IU/dL), neither protein C activity below nor above the laboratory reference range was significantly associated with stroke severity or poor functional outcome compared with the within-reference-range group (Supplementary Table 9). Furthermore, this variable was not independently associated with either initial stroke severity or poor functional outcome (Supplementary Table 9), suggesting that the observed associations are unlikely to be explained solely by categorical protein C deficiency.

Ordinal logistic regression demonstrated an inverse association between protein C activity and worsening functional outcome across the full 7-level mRS (Supplementary Table 10). The association was strongest for the second and third protein C activity quartiles, whereas the highest quartile was not significantly associated with functional outcome, consistent with the primary binary logistic regression analysis.

No statistically significant interaction between age and protein C activity was observed for moderate-to-severe stroke (P for interaction = 0.72) (Supplementary Table 11).

In secondary analyses, adjustment for atrial fibrillation markedly attenuated the association between protein C activity and cardioembolic stroke, whereas the association between protein C activity and stroke severity remained significant (Supplementary Tables 12, 13).

## 4 Discussion

Lower protein C activity was consistently associated with greater initial stroke severity. An association with poor functional outcome was also observed in the primary analyses, although this association was less consistent across sensitivity analyses and demonstrated a nonlinear relationship. Notably, conventional definitions of protein C deficiency were not associated with outcomes, suggesting that graded variation in protein C activity, rather than categorical deficiency alone, may be clinically relevant. In addition, lower protein C activity was associated with a higher prevalence of atrial fibrillation and cardioembolic stroke, supporting a link between endogenous anticoagulant activity and thrombogenic mechanisms.

Although aging is generally associated with a shift toward a prothrombotic state (Franchini, 2006; Mari et al., 2008; North and Sinclair, 2012), we did not detect statistically significant effect modification by age in the present study. However, the absence of a statistically significant interaction should not be interpreted as evidence that age has no modifying effect. Rather, these findings indicate that no statistically significant interaction between age and protein C activity was detected for stroke severity within the statistical power of the present cohort. Larger studies with greater statistical power are warranted to determine whether age modifies the association between protein C activity and stroke severity.

Taken together, these findings suggest that variation in protein C activity across the observed distribution may reflect differences in endogenous anticoagulant function as well as other biological processes relevant to thrombogenic vulnerability. Additional analyses based on conventional laboratory reference categories yielded similar overall interpretations, suggesting that clinically relevant variation in protein C activity may not be fully captured by predefined laboratory thresholds (Folsom et al., 2009). Because protein C activity was measured after stroke onset, reduced protein C activity may partly reflect activation of coagulation and inflammatory pathways triggered by more severe cerebral ischemia rather than representing a causal determinant of stroke severity. Although protein C activity was measured at hospital admission before substantial in-hospital treatment and prior to clinical outcome assessment, and our analyses adjusted for baseline stroke severity and other major confounders, residual reverse causality cannot be completely excluded. Therefore, our findings should be interpreted as demonstrating an association rather than causation. Future prospective studies incorporating serial protein C measurements before and after stroke onset are warranted to clarify the temporal relationship and causal mechanisms.

However, protein C activity measured during the acute phase of ischemic stroke may also be influenced by acute illness, systemic inflammation, hepatic synthetic function, vitamin K status, anticoagulant therapy, and other comorbid conditions. Therefore, protein C activity should be interpreted as an integrated biomarker reflecting multiple physiological processes rather than as a direct measure of endogenous anticoagulant function alone. Protein C plays a central role in regulating thrombin generation, and reduced activity may contribute to a prothrombotic state associated with more severe ischemic injury. Rather than representing a simple binary deficiency state, variation in protein C activity across the observed distribution may capture clinically meaningful biological heterogeneity associated with thrombotic vulnerability. Our findings further suggest that conventional laboratory reference intervals may not necessarily represent the optimal biological thresholds for risk stratification in acute ischemic stroke.

Consistent with this interpretation, restricted cubic spline analyses did not demonstrate evidence of a nonlinear association between protein C activity and initial stroke severity, whereas a nonlinear relationship was observed for functional outcome (Anzola et al., 1993; Martinez et al., 1993; Dahlbäck, 1995). The lack of a strictly monotonic association across protein C activity quartiles, together with the nonlinear pattern identified by the spline analysis, suggests that the relationship between protein C activity and functional outcome may not be adequately characterized by a simple linear dose–response model.

The observed association between lower protein C activity and a higher prevalence of atrial fibrillation and cardioembolic stroke is consistent with a potential link between endogenous anticoagulant pathways and AF-related thrombogenesis. The protein C pathway regulates thrombin generation through interactions among thrombin, thrombomodulin, and the endothelial protein C receptor (Anzola et al., 1993; Negreva et al., 2017). Structural remodeling and endothelial dysfunction in atrial fibrillation may impair activation of this pathway and promote a prothrombotic state (Minuk et al., 2010; Kovacs et al., 2006). After adjustment for atrial fibrillation, the association between protein C activity and cardioembolic stroke was attenuated and no longer statistically significant, suggesting that atrial fibrillation may partly account for the observed relationship. However, because no formal mediation analysis was performed, these findings should not be interpreted as evidence of a causal mediating effect. Notably, the association between protein C activity and stroke severity remained significant after adjustment for atrial fibrillation, suggesting that protein C activity may reflect broader thrombogenic or microcirculatory mechanisms beyond AF-related embolism. Furthermore, sensitivity analyses performed without adjustment for TOAST subtype yielded findings that were materially consistent with the primary analyses, suggesting that the observed associations between protein C activity and clinical outcomes were not solely dependent on adjustment for stroke subtype. Similar findings were observed in the ordinal logistic regression shift analysis using the full 7-level mRS, suggesting that the association between protein C activity and functional outcome was not dependent on dichotomization of the outcome.

Our findings also provide context for previous studies examining protein C and arterial thrombosis. Early clinical reports suggested that inherited protein C deficiency might contribute to ischemic stroke, particularly in younger patients (Anzola et al., 1993; Camerlingo et al., 1991). However, subsequent population-based studies have reported inconsistent associations between protein C levels and arterial thrombotic events. For example, the ARIC study reported an association between lower protein C levels and incident ischemic stroke (Folsom et al., 2009), whereas the REGARDS study observed only modest associations with cardiovascular outcomes and no clear relationship with stroke risk (Zakai et al., 2018). The present findings suggest that examining protein C activity across graded distributions may provide more informative insights than relying solely on categorical deficiency thresholds.

From a clinical perspective, protein C activity is routinely measurable at hospital admission and may provide additional information regarding stroke severity and the underlying biological processes associated with AIS. However, the present study did not assess incremental predictive performance or establish clinical utility.

This study has several limitations. First, as an observational study, causal relationships cannot be established, and residual confounding may remain despite multivariable adjustment. Second, protein C activity was measured only once at the time of hospital admission and therefore may not fully capture temporal variation during the acute phase. Although blood sampling was standardized at admission, patients presented at different times within 7 days after symptom onset, and thus the interval between stroke onset and blood sampling was not uniform across the cohort. Third, this was a single-center study, which may limit generalizability. Fourth, despite adjustment for baseline stroke severity and major clinical confounders, reverse causality cannot be completely excluded because protein C activity was measured after stroke onset (Minuk et al., 2010; Kovacs et al., 2006). Fifth, although 3-month functional outcome data were unavailable for a proportion of patients, patients without available follow-up had lower protein C activity and higher frequencies of female sex, hypertension, diabetes mellitus, and dyslipidemia than those with available follow-up. These findings suggest that loss to follow-up was not completely random and may have introduced residual bias despite the use of IPW. Additional sensitivity analyses demonstrated that the association between protein C activity and functional outcome was less consistent than the association with initial stroke severity. Furthermore, the restricted cubic spline suggested a nonlinear relationship rather than a simple graded inverse dose–response pattern. Accordingly, the association between protein C activity and functional outcome should be interpreted with appropriate caution. Some degree of residual bias due to incomplete follow-up, however, cannot be completely excluded. Finally, infarct volume, large-vessel occlusion status, premorbid functional status, post-stroke treatment, and rehabilitation data were not consistently available in our registry. These factors may independently influence functional outcome and therefore represent potential sources of residual confounding.

Although protein C activity was independently associated with stroke severity and functional outcome, the present study was designed to evaluate associations rather than incremental predictive performance. Therefore, the current findings should not be interpreted as evidence supporting the clinical use of protein C activity for risk stratification. Future prospective studies should determine whether protein C activity provides incremental predictive value beyond established clinical predictors before its incorporation into routine clinical practice can be considered.

Lower protein C activity was consistently associated with greater initial stroke severity across multiple analyses. In contrast, the association with poor functional outcome was less consistent across the additional sensitivity analyses and should therefore be interpreted cautiously. Further prospective studies are warranted to better characterize the association between protein C activity and long-term functional outcomes.

## Data Availability

The original contributions presented in the study are included in the article/Supplementary material, further inquiries can be directed to the corresponding author.
