# Supplementary material for: Protein C activity may reflect endogenous anticoagulant balance and is associated with stroke severity and functional outcomes in acute ischemic stroke
Source: Front Aging Neurosci. 2026 Aug 7;18:1872982. doi: 10.3389/fnagi.2026.1872982 (PMC13493530; doi:10.3389/fnagi.2026.1872982)
Supplement: Supplementary file 1 [file Data_Sheet_1.DOCX]

Supplementary Material

**Supplementary Figure 1. Flow diagram of patient selection.**

Of the 1,859 patients enrolled in the prospective institutional stroke registry, only patients with acute ischemic stroke (AIS) were eligible for inclusion in the present study. Patients with transient ischemic attack (TIA) and other non-AIS diagnoses were excluded before cohort selection. After excluding 45 patients without available protein C activity measurements, the final analytic cohort comprised 1,083 patients. Three-month modified Rankin Scale (mRS) scores were available for 865 patients, whereas 218 patients had missing follow-up outcome data.

**Supplementary Table 1. Continuous association between protein C activity and clinical outcomes**

| Outcome | Predictor | Adjusted OR | 95% CI | *P*-value |
| --- | --- | --- | --- | --- |
| Moderate-to-severe stroke (NIHSS ≥5) | Protein C activity (per 1-SD increase) | 0.63 | 0.56–0.72 | <0.001 |
| Poor functional outcome (3-month mRS ≥3) | Protein C activity (per 1-SD increase) | 0.71 | 0.61–0.82 | <0.001 |

OR, Odds Ratio; CI, confidence interval; mRS, modified Rankin Scale; NIHSS, National Institutes of Health Stroke Scale

**Supplementary Table 2. Analysis of Multivariable Logistic Regression: Evaluating the Influence of protein C references on Initial neurological severity (initial NIHSS 0-4 versus ≥5)**

| Variables | | OR | 95% CI | *P*-value |
| --- | --- | --- | --- | --- |
| Age, per 1 years | | 1.021 | 1.008-1.034 | 0.002 |
| Female, sex | | 1.286 | 0.907-1.825 | 0.158 |
| TOAST classification | |  |  |  |
| Large artery atherosclerosis | | Reference |  |  |
| Small vessel occlusion | | 0.503 | 0.323-0.784 | 0.002 |
| Cardioembolic | | 0.858 | 0.488-1.510 | 0.596 |
| Other determined | | 1.205 | 0.575-2.524 | 0.621 |
| Undetermined, 2 or more | | 1.004 | 0.542-1.859 | 0.990 |
| Negative | | 1.402 | 0.777-2.531 | 0.262 |
| incomplete | | 1.335 | 0.739-2.411 | 0.339 |
| Coronary heart disease history | | 1.067 | 0.703-1.736 | 0.665 |
| Cardiovascular risk factor | |  |  |  |
| Dyslipidemia | | 0.673 | 0.446-1.016 | 0.060 |
| Smoking history | | 1.264 | 0.863-1.852 | 0.228 |
|  | Atrial fibrillation | 2.203 | 1.308-3.712 | 0.003 |
| Laboratory findings | |  |  |  |
| Hematocrit | | 1.021 | 0.999-1.044 | 0.063 |
| Blood Urea Nitrogen | | 1.005 | 0.989-1.021 | 0.554 |
| C-reactive protein | | 1.020 | 1.012-1.029 | <0.001 |
| Protein C activity, normal (65-135) | | Reference |  |  |
| Low (<65) | | 1.319 | 0.689-2.524 | 0.404 |
| High (>135) | | 0.637 | 0.377-1.078 | 0.093 |

OR, Odds Ratio; CI, confidence interval; TOAST, Trial of ORG 10172 in Acute Stroke Treatment

Adjusted for age, sex, hypertension, diabetes mellitus, stroke subtype (TOAST classification), coronary heart disease history, dyslipidemia, smoking history, atrial fibrillation, hematocrit, blood urea nitrogen, and C-reactive protein.

**Supplementary Table 3. Analysis of Multivariable Logistic Regression: Evaluating the Influence of protein C references on functional outcomes (3-month mRS 0-2 versus ≥3)**

| Variables | OR | 95% CI | *P*-value |
| --- | --- | --- | --- |
| Age, per 1 years | 1.050 | 1.031-1.068 | <0.001 |
| Female, sex | 1.267 | 0.834-1.924 | 0.267 |
| TOAST classification |  |  |  |
| Large artery atherosclerosis | Reference |  |  |
| Small vessel occlusion | 0.807 | 0.480-1.357 | 0.419 |
| Cardioembolic | 0.350 | 0.167-0.735 | 0.006 |
| Other determined | 0.642 | 0.245-1.679 | 0.366 |
| Undetermined, 2 or more | 1.026 | 0.479-2.199 | 0.947 |
| Negative | 0.610 | 0.289-1.289 | 0.195 |
| Incomplete | 0.956 | 0.452-2.020 | 0.906 |
| Cardiovascular risk factor |  |  |  |
| Coronary heart disease history | 1.345 | 0.793-2.284 | 0.272 |
| Dyslipidemia | 1.236 | 0.768-1.990 | 0.383 |
| Smoking history | 0.880 | 0.545-1.422 | 0.602 |
| Atrial fibrillation | 1.985 | 1.001-3.934 | 0.050 |
| Laboratory findings |  |  |  |
| Hematocrit | 1.011 | 0.986-1.037 | 0.398 |
| Blood Urea Nitrogen | 1.010 | 0.992-1.028 | 0.299 |
| C-reactive protein | 1.011 | 1.003-1.019 | 0.008 |
| Protein C activity, normal (65-135) | reference |  |  |
| Low (<65) | 1.948 | 0.877-4.325 | 0.101 |
| High (>135) | 1.761 | 0.994-3.121 | 0.053 |
| Initial Stroke Severity, NIHSS 0-4 | reference |  |  |
| NIHSS ≥5 | 5.598 | 3.848-8.144 | <0.001 |

OR, Odds Ratio; CI, confidence interval; NIHSS, National Institutes of Health Stroke Scale; TOAST, Trial of ORG 10172 in Acute Stroke Treatment

Adjusted for age, sex, hypertension, diabetes mellitus, stroke subtype (TOAST classification), coronary heart disease history, dyslipidemia, smoking history, atrial fibrillation, hematocrit, blood urea nitrogen, C-reactive protein, and initial NIHSS.

**Supplementary Table 4.** Comparison of baseline characteristics between patients with and without available 3-month functional outcome data

| Variable | 3-month mRS available  (n= 865) | Missing 3-month mRS  (n= 218) | *P*-value |
| --- | --- | --- | --- |
| Age, mean ± SD | 68.6 ± 13.4 | 68.7 ± 14.0 | 0.959 |
| Protein C activity, mean ± SD | 104.8 ± 26.3 | 98.0 ± 25.8 | <0.001 |
| Initial NIHSS, mean ± SD | 4.6 ± 5.3 | 4.4 ± 6.2 | 0.667 |
| Female, n (%) | 353 (40.8) | 110 (50.5) | 0.010 |
| Hypertension, n (%) | 544 (63.0) | 169 (77.5) | <0.001 |
| Diabetes, n (%) | 290 (33.5) | 96 (44.0) | 0.004 |
| Dyslipidemia, n (%) | 158 (18.3) | 59 (27.1) | 0.004 |
| Smoking, n (%) | 259 (30.0) | 71 (32.6) | 0.451 |
| Atrial fibrillation, n (%) | 162 (18.7) | 51 (23.4) | 0.121 |
| Previous anticoagulation, n (%) | 36 (4.2) | 15 (6.9) | 0.090 |

NIHSS, National Institutes of Health Stroke Scale

**Supplementary Table 5. Inverse probability weighting (IPW) sensitivity analysis for poor functional outcome (3-month mRS ≥3)**

| Variable | OR | 95% CI | *P*-value |
| --- | --- | --- | --- |
| Protein C activity quartiles |  |  |  |
| Q1 | Reference |  |  |
| Q2 | 0.49 | 0.29–0.83 | 0.008 |
| Q3 | 0.53 | 0.31–0.92 | 0.024 |
| Q4 | 0.94 | 0.55–1.60 | 0.811 |
| Protein C activity (per 1-SD increase) | 0.99 | 0.80–1.21 | 0.898 |

OR, Odds Ratio; CI, confidence interval; IPW, Inverse probability weighting; mRS, modified Rankin Scale

Odds ratios were estimated using the same multivariable logistic regression model as the primary analysis, with additional inverse probability weighting to account for potential bias due to incomplete follow-up.

**Supplementary Table 6. Sensitivity analyses after additional adjustment for reperfusion therapy**

| **Outcome** | Variable | OR | **95% CI** | *P*-value |
| --- | --- | --- | --- | --- |
| Initial stroke severity  (NIHSS ≥5) | |  |  |  |
|  | Protein C activity quartiles |  |  |  |
|  | Q1 | Reference |  |  |
|  | Q2 | 0.60 | 0.38–0.93 | 0.022 |
|  | Q3 | 0.66 | 0.42–1.05 | 0.078 |
|  | Q4 | 0.57 | 0.35–0.99 | 0.025 |
| Poor functional outcome  (3-month mRS ≥3) | |  |  |  |
|  | Protein C activity quartiles |  |  |  |
|  | Q1 | Reference |  |  |
|  | Q2 | 0.76 | 0.46–1.28 | 0.305 |
|  | Q3 | 0.60 | 0.35–1.03 | 0.066 |
|  | Q4 | 1.28 | 0.74–2.20 | 0.379 |

OR, odds ratio; CI, confidence interval; NIHSS, National Institutes of Health Stroke Scale; mRS, modified Rankin Scale.

Models were additionally adjusted for reperfusion therapy (intravenous thrombolysis and/or endovascular thrombectomy) in addition to the covariates included in the corresponding primary multivariable models. Initial NIHSS was additionally included only in the functional outcome model.

**Supplementary Table 7. Sensitivity analyses after excluding patients receiving pre-admission anticoagulant therapy**

| **Outcome** | Variable | OR | **95% CI** | *P*-value |
| --- | --- | --- | --- | --- |
| Initial stroke severity  (NIHSS ≥5) |  |  |  |  |
|  | Protein C activity quartiles |  |  |  |
|  | Q1 | Reference |  |  |
|  | Q2 | 0.66 | 0.43–1.02 | 0.061 |
|  | Q3 | 0.57 | 0.37–0.89 | 0.014 |
|  | Q4 | 0.58 | 0.36–0.92 | 0.020 |
| Poor functional outcome (3-month mRS ≥3) |  |  |  |  |
|  | Protein C activity quartiles |  |  |  |
|  | Q1 | Reference |  |  |
|  | Q2 | 0.53 | 0.30–0.93 | 0.026 |
|  | Q3 | 0.57 | 0.33–0.99 | 0.045 |
|  | Q4 | 0.99 | 0.56–1.74 | 0.969 |

OR, Odds Ratio; CI, confidence interval; mRS, modified Rankin Scale

Models were adjusted for the same covariates as the corresponding primary multivariable analyses after excluding patients receiving pre-admission anticoagulant therapy. Initial NIHSS was additionally included only in the functional outcome model.

**Supplementary Table 8. Sensitivity analyses without adjustment for TOAST subtype**

| **Outcome** | **Value** | **OR** | **95% CI** | *P*-value |
| --- | --- | --- | --- | --- |
| Moderate-to-severe stroke (NIHSS ≥5) |  |  |  |  |
|  | Protein C activity quartiles |  |  |  |
|  | Q1 | Reference |  |  |
|  | Q2 | 0.70 | 0.46–1.05 | 0.086 |
|  | Q3 | 0.55 | 0.36–0.85 | 0.007 |
|  | Q4 | 0.57 | 0.37–0.88 | 0.012 |
| Poor functional outcome  (3-month mRS ≥3) |  |  |  |  |
|  | Protein C activity quartiles |  |  |  |
|  | Q1 | Reference |  |  |
|  | Q2 | 0.52 | 0.32–0.84 | 0.008 |
|  | Q3 | 0.52 | 0.32–0.85 | 0.008 |
|  | Q4 | 0.82 | 0.50–1.35 | 0.437 |

OR, Odds Ratio; CI, confidence interval; NIHSS, National Institutes of Health Stroke Scale; TOAST, Trial of ORG 10172 in Acute Stroke Treatment

Models were adjusted for age, sex, hypertension, diabetes mellitus, coronary heart disease history, dyslipidemia, smoking history, atrial fibrillation, hematocrit, blood urea nitrogen, and C-reactive protein. TOAST subtype was not included in these models. Initial NIHSS was additionally included only in the functional outcome model.

**Supplementary Table 9.** **Associations between protein C activity categories based on the laboratory reference range and clinical outcomes**

| **Outcome** | **Protein C category** | **OR** | **95% CI** | *P*-value |
| --- | --- | --- | --- | --- |
| Initial stroke severity (NIHSS ≥5) | 65–135 IU/dL | Reference |  |  |
|  | <65 IU/dL | 1.33 | 0.71–2.50 | 0.373 |
|  | >135 IU/dL | 0.62 | 0.37–1.05 | 0.076 |
| Poor functional outcome (3-month mRS ≥3) | 65–135 IU/dL | Reference |  |  |
|  | <65 IU/dL | 1.60 | 0.71–3.58 | 0.257 |
|  | >135 IU/dL | 1.67 | 0.96–2.91 | 0.072 |

OR, odds ratio; CI, confidence interval; NIHSS, National Institutes of Health Stroke Scale; mRS, modified Rankin Scale.

Protein C activity was categorized as below (<65 IU/dL), within (65–135 IU/dL), or above (>135 IU/dL) the laboratory reference range. The within-reference-range category was used as the reference. Models were adjusted for the same covariates as the corresponding primary analyses; initial NIHSS was additionally included only in the model evaluating 3-month functional outcome.

**Supplementary Table 10. Ordinal logistic regression (proportional odds model) using the full 7-level modified Rankin Scale**

| Variable | Common OR | 95% CI | *P*-value |
| --- | --- | --- | --- |
| Protein C activity quartiles |  |  |  |
| Q1 | Reference |  |  |
| Q2 | 0.63 | 0.44–0.91 | 0.014 |
| Q3 | 0.53 | 0.36–0.77 | <0.001 |
| Q4 | 0.87 | 0.59–1.29 | 0.486 |

OR, Odds Ratio; CI, confidence interval; NIHSS, National Institutes of Health Stroke Scale; TOAST, Trial of ORG 10172 in Acute Stroke Treatment

Ordinal logistic regression (proportional odds model) was performed using the full 7-level modified Rankin Scale (0–6) at 3 months as the outcome variable. The model was adjusted for age, sex, hypertension, diabetes mellitus, stroke subtype (TOAST classification), coronary heart disease history, dyslipidemia, smoking status, atrial fibrillation, hematocrit, blood urea nitrogen, C-reactive protein, and initial NIHSS. The proportional-odds assumption was evaluated and was not violated.

**Supplementary Table 11. Interaction and age-stratified analyses of the association between continuous protein C activity and moderate-to-severe stroke (NIHSS ≥5)**

| Analysis | OR | 95% CI | *P*-value |
| --- | --- | --- | --- |
| Protein C × Age interaction | 0.99 | 0.97–1.02 | 0.72 |
| Age-stratified analyses |  |  |  |
| <65 years | 1.06 | 0.91–1.24 | 0.43 |
| ≥65 years | 0.94 | 0.87–1.02 | 0.12 |
| <75 years | 1.01 | 0.94–1.08 | 0.82 |
| ≥75 years | 0.96 | 0.88–1.05 | 0.41 |

OR, Odds Ratio; CI, confidence interval; NIHSS, National Institutes of Health Stroke Scale

Outcome: Moderate-to-severe stroke (NIHSS ≥5).

Predictor: Protein C activity analyzed as a continuous variable (per 1-SD increase).

Interaction analysis: A protein C × age interaction term was included in the multivariable logistic regression model.

Age-stratified analyses: Separate multivariable logistic regression models were fitted for participants aged <65 and ≥65 years and <75 and ≥75 years.

Models were adjusted for the same covariates as the primary multivariable analysis.

**Supplementary Table 12. Association between protein C activity and cardioembolic stroke before and after adjustment for atrial fibrillation**

| Model | OR | 95% CI | *P*-value |
| --- | --- | --- | --- |
| Model 1: Age + sex adjusted |  |  |  |
| Protein C activity (per quartile increase) | 0.72 | 0.61–0.84 | <0.001 |
|  |  |  |  |
| Model 2: Age + sex + atrial fibrillation adjusted |  |  |  |
| Protein C activity (per quartile increase) | 1.00 | 0.82–1.22 | 0.99 |
| Atrial fibrillation | 26.34 | 17.04–40.73 | <0.001 |

CI, confidence interval; OR, Odds Ratio

**Supplementary Table 13. Association between protein C activity and moderate-to-severe stroke (NIHSS ≥5) after adjustment for atrial fibrillation**

| Variable | OR | 95% CI | *P*-value |
| --- | --- | --- | --- |
| Protein C activity (per quartile increase) | 0.76 | 0.67–0.86 | <0.001 |
| Age (per year) | 1.03 | 1.02–1.04 | <0.001 |
| Atrial fibrillation | 2.32 | 1.66–3.22 | <0.001 |

OR, Odds Ratio
